# Whole-genome sequences of six *Borrelia recurrentis* strains obtained via PacBio sequencing

**DOI:** 10.1128/mra.01026-24

**Published:** 2025-01-13

**Authors:** Alhussien M. Gaber, John C. Blazier, Artem S. Rogovskyy

**Affiliations:** 1 Department of Pathobiology and Diagnostic Investigation, College of Veterinary Medicine, Michigan State University, East Lansing, Michigan, USA; 2 Department of Veterinary Pathobiology, College of Veterinary Medicine and Biomedical Sciences, Texas A&M University, College Station, Texas, USA; 3 Texas A&M Institute for Genomics Sciences and Society, Texas A&M University, College Station, Texas, USA; The University of Arizona, Tucson, Arizona, USA

**Keywords:** Borrelia, whole genome, sequences, louse-borne relapsing fever

## Abstract

Provided are whole-genome sequences of six *Borrelia recurrentis* strains that had been earlier isolated from louse-borne relapsing fever patients. The sequences of each genome presented here included one linear chromosome and 5 linear plasmids, whose average size was 1,284,895 bp with the mean GC content being 27.5%.

## ANNOUNCEMENT

Louse-borne relapsing fever (LBRF) is a vector-borne disease whose morbidity and mortality are significant in some African countries (1–5). LBRF is caused by *Borrelia recurrentis*, the spirochetal bacterium that affects only humans with no known animal reservoir (5, 6). The genome of *B. recurrentis* strain A1 previously obtained by shotgun approach was shown to comprise a 0.9 Mb chromosome and 7 linear plasmids (lp124, lp53, lp37, lp35, lp33, lp23, lp6) (7). A more recent study that utilized Illumina MiSeq technology to sequence six additional *B. recurrentis* strains (A17, PBeK, PAbJ, PAbN, PMaC, PUfA) demonstrated almost identical genome compositions (8). The exception was lp124, whose size was consistently larger for the six strains (8), supporting previously generated pulsed-field gel electrophoresis data (9–12). This discrepancy, and an inherent failure of both sequencing methods to accurately define the *B. recurrentis* antigenic variation system because of its highly homologous elements (13), prompted us to re-sequence strains A1, A11, A17, PBeK, PAbJ, and PAbN by using the long-read PacBio technology.

The origins of strains are as follow. The A strains were isolated from the blood of LBRF patients originated from Ethiopia in the 1990s (9, 10). PBeK and PAbN were isolated in Germany in 2004 and 2015 from one LBRF patient returning and the other migrating from Ethiopia, respectively (8). PAbJ was isolated in Germany in 2015 from a LBRF patient originated from Somali (8).

*B. recurrentis* was propagated to ~5 × 10^7^–1 × 10^8^ cells/mL in home-made Barbour-Stoenner-Kelly II medium supplemented with 10% (vol/vol) rabbit serum (Sigma-Aldrich, MO, USA) and 1.4% (wt/vol) gelatin (Sigma-Aldrich, MO, USA) at 35°C under 2.5% CO_2_ (14), and pellets were shipped to Maryland Genomics, Institute for Genome Sciences (IGS), University of Maryland School of Medicine (MD, Baltimore, USA) for DNA extraction and sequencing. DNA samples were extracted using the Qiagen MagAtract HMW DNA kit (Qiagen, MI, USA) and sheared to an average size of 10–15 kb. Libraries prepared using the SMRTbell Express Template Prep Kit 2.0 (Pacific Biosciences, CA, USA) were size-selected on the BluePippin instrument (Sage Science, MA, USA) to remove fragments of <10 kb, which excluded the smallest previously sequenced plasmid, lp6 (7, 8). The library pool was sequenced with Sequel II Sequencing Kit 2.0 and 8M SMRT cell on the Sequel II instrument (Pacific Bioscience, CA, USA).

De-novo assemblies were performed using IPA (SMRTtools version 11.0.0) at IGS. On the Grace computing cluster at Texas A&M University, PacBio reads were downsampled to 25K sequences per sample using seqtk (version 1.3) due to the extremely high coverage of the data set (Table 1). The downsampled data were then assembled in Flye (version 2.9.1) under default parameters. Contigs were annotated using the NCBI Prokaryotic Genome Annotation Pipeline (15).

**TABLE 1:** Statistics of *de novo* assembled contigs and genome composition of *Borrelia recurrentis* strains A1, A11, A17, PAbJ, PBeK, and PAbN

	*Borrelia recurrentis* strains^*a*^					
	A1	A11	A17	PAbJ	PBeK	PAbN
Contig assembly statistics						
Total bases	4,652,507,565	3,108,737,453	5,393,071,144	4,434,757,658	4,936,239,489	4,851,771,615
Number of reads	370,257	254,752	421,692	363,105	393,574	393,519
N50 read length	12,594	12,242	12,809	12,257	12,567	12,338
N50 contig length	932,334	932,334	932,354	932,347	932,332	932,342
Average coverage (X)	3,639	2,410	4,199	3,440	3,844	3,778
Total DNA size	1,278,199	1,289,858	1,284,120	1,288,942	1,284,107	1,284,144
Genome features						
Chromosome	932,334	932,334	932,354	932,347	932,332	932,342
lp190^*b*^	190,003	190,002	190,010	189,996	189,995	189,998
lp55	55,036	55,033	55,035	55,042	55,042	55,045
lp42	42,100	42,085	42,100	42,120	42,116	42,121
lp35	35,472	35,483	35,480	35,475	35,475	35,483
lp23	23,254	34,921	29,141	33,962	29,147	29,155
Total genes	1,260	1,275	1,268	1,272	1,268	1,265
Total GC content (%)	27.4	27.5	27.5	27.5	27.5	27.5
SRA accession numbers	SRX26214741	SRX26214742	SRX26214743	SRX26214744	SRX26214745	SRX26214746
Gene bank accession numbers	CP169982-CP169987	CP169976-CP169981	CP169970-CP169975	CP169964-CP169969	CP169958-CP169963	CP169952-CP169957

^*a*^ Sizes are given in base pair (bp).

^*b*^ lp denotes linear plasmid. The numbers consistently indicate the respective plasmid size in kb for all six strains except for lp23, whose size varies between the strains. The smallest plasmid, lp6 was excluded from the analysis.

Each assembled *B. recurrentis* genome contained one chromosome and 5 linear plasmids (Table 1). To determine the completeness of all chromosomes and plasmids, we identified *B. recurrentis*-specific telomeric box 3 sequences (5′-TTAGTATA-3′ and 5′-TATACTAA-3′), which are conserved inverted terminal repeats recognized by telomere resolvase during borrelial replication (16–19). As a result, previously sequenced lp124 and lp37 of strain A1 (7) were found to constitute a single plasmid, lp190. Overall, the present data demonstrated that the *B. recurrentis* chromosomes and four largest plasmids, lp190, lp55, lp42, and lp35, had similar lengths in all the strains, whereas lp23 size varied considerably between the six strains.

## Data Availability

The whole genome sequences of *B. recurrentis* strains A1, A11, A17, PAbJ, PBeK, and PAbN were deposited in the GenBank database under the following accession numbers: CP169982-CP169987, CP169976-CP169981, CP169970-CP169975, CP169964-CP169969, CP169958-CP169963, and CP169952-CP169957, respectively (Table 1). Raw reads have been deposited in the Sequence Read Archive under the following accession numbers: SRX26214741, SRX26214742, SRX26214743, SRX26214744, SRX26214745, and SRX26214746 (Table 1). The BioProject accession number is PRJNA1157803. The version described in this paper is the first version.
